# Estimating the SARS-CoV2 infections detection rate and cumulative incidence in the World Health Organization African Region 10 months into the pandemic

**DOI:** 10.1017/S0950268821002417

**Published:** 2021-11-04

**Authors:** Benido Impouma, Franck Mboussou, Cyrus Shahpar, Caitlin M. Wolfe, Bridget Farham, George Sie Williams, Humphrey Karamagi, Roland Ngom, Ngoy Nsenga, Antoine Flahault, Cláudia Torres Codeço, Zabulon Yoti, Francis Kasolo, Olivia Keiser

**Affiliations:** 1 World Health Organization, Regional Office for Africa, Brazzaville, Congo; 2 Resolve to Save Lives, New York, New York, USA; 3 College of Public Health, University of South Florida, Tampa, Florida, USA; 4 Institute of Global Health, University of Geneva, Geneva, Switzerland; 5 Fundação Oswaldo Cruz, Rio de Janeiro, Brazil

**Keywords:** COVID-19, cumulative incidence, detection rate, SARS-CoV-2

## Abstract

As of 03 January 2021, the WHO African region is the least affected by the coronavirus disease-2019 (COVID-19) pandemic, accounting for only 2.4% of cases and deaths reported globally. However, concerns abound about whether the number of cases and deaths reported from the region reflect the true burden of the disease and how the monitoring of the pandemic trajectory can inform response measures.

We retrospectively estimated four key epidemiological parameters (the total number of cases, the number of missed cases, the detection rate and the cumulative incidence) using the COVID-19 prevalence calculator tool developed by Resolve to Save Lives. We used cumulative cases and deaths reported during the period 25 February to 31 December 2020 for each WHO Member State in the region as well as population data to estimate the four parameters of interest. The estimated number of confirmed cases in 42 countries out of 47 of the WHO African region included in this study was 13 947 631 [95% confidence interval (CI): 13 334 620–14 635 502] against 1 889 512 cases reported, representing 13.5% of overall detection rate (range: 4.2% in Chad, 43.9% in Guinea). The cumulative incidence of severe acute respiratory syndrome coronavirus-2 (SARS-CoV-2) was estimated at 1.38% (95% CI: 1.31%–1.44%), with South Africa the highest [14.5% (95% CI: 13.9%–15.2%)] and Mauritius [0.1% (95% CI: 0.099%–0.11%)] the lowest. The low detection rate found in most countries of the WHO African region suggests the need to strengthen SARS-CoV-2 testing capacities and adjusting testing strategies.

## Key results:

- Detection rate of COVID-19 cases in the WHO African region is estimated at 13.5%, ranging from 4.2% to 43.9%.
- Cumulative incidence of SARS-CoV2 infection in the WHO African estimated at 1.38%.
- South Africa was the country with the highest estimated incidence (14.5%) and Mauritius the lowest (0.1%).

## Introduction

In late December 2019, a novel coronavirus identified as severe acute respiratory syndrome coronavirus-2 (SARS-CoV-2) was detected in a cluster of pneumonia cases reported in Wuhan City, Hubei Province, China [1]. Within a month, the disease, named by the World Health Organization (WHO) as coronavirus disease-2019 (COVID-19), spread rapidly across international borders and was declared a pandemic by the WHO on 11 March 2020 [2].

The first case of COVID-19 was reported in the WHO African region, the last of the six WHO regions to be affected, on 25 February 2020 in a traveller in Algeria [3]. With fragile health systems, limited testing capacities and potentially vulnerable populations, it was anticipated that countries in the WHO African region would be among the worst affected by the COVID-19 pandemic [4]. One model estimated over 223 million cases and 150 000 deaths in the WHO African region by the end of 2020, assuming widespread community transmission [5]. By 3 January 2021, the region remained among the least affected accounting for 2.4% (*n* = 1.9 million out of 83.3 million) and 2.4% (*n* = 43 600 out of 1.8 million) of globally reported COVID-19 cases and deaths, respectively [6]. However, there are concerns that the true number of COVID-19 cases in the region may be higher than being reported, largely due to limited testing capacities affecting the detection rate across countries in the WHO African region [7]. Additionally, the high proportion of asymptomatic and mild cases being reported amidst the reluctance of the population to seek testing services or report to health facilities reduce utilisation of testing services, contributing to underestimation of the burden of the infection [8].

The actual number of infected persons in the region is likely higher than reported, making epidemic monitoring difficult and undermining efforts to control it. Understanding the true burden of SARS-CoV-2 infection can help countries not only monitor the effect of the pandemic but also adopt customised approaches and response actions to mitigate and control the pandemic. Seroprevalence studies would provide a more accurate picture [9], which can be used to estimate the true number of SARS-CoV-2 infections. The estimated cumulative incidence of SARS-CoV-2 infections can provide a measure of the extent to which transmission of the disease has occurred in the population [9]. While such a study when designed appropriately can offer estimates of these metrics, a nationwide implementation may be constrained by competing with immediate priorities of the ongoing outbreak response, extensive resource requirements and the need for repeated cross-sectional analyses tracking seroprevalence to more completely measure the true incidence of SARS-CoV-2 [10]. Cost-effective and simplified approaches, which provide reliable estimates of these metrics at a larger population level, are needed in the WHO African region. The objective of this study, therefore, is to derive estimates of key metrics for determining the actual extent of SARS-CoV-2 infections in the Member States in the WHO African region using an alternate approach that is real-time, simplified and cost-effective. Specifically, the study aims to estimate the cumulative incidence of SARS-CoV2 infections and detection rate in all countries of the WHO African region, analyse the implications of the estimates on the pandemic dynamics and provide guidance to countries on actions that could be taken using these metrics.

## Methods

We retrospectively analysed COVID-19 confirmed cases and deaths reported from the WHO Member States in the African region from 25 February to 31 December 2020 along with population data to estimate key measures of the burden and severity of the disease in the region. The WHO African region consists of 47 Member States mostly in sub-Sahara Africa [11] with a combined estimated population of 1.1 billion in 2019 [12].

### Data sources and measurement

We used the cumulative number of COVID-19 cases and deaths, the estimated population in 2019 [12] in each Member State and an empirical infection fatality ratio (IFR) from available literature to estimate the true number of COVID-19 cases, the detection rate and the cumulative incidence. The WHO Regional Office for Africa maintains a line list of COVID-19 cases and deaths routinely reported by the Member States. These reports served as the data sources for the cumulative number of cases and deaths. The population was stratified by two age groups, 65 years and above and those below 65 years, in line with reported age-associated COVID-19 mortality [13, 14]. Population estimates for each Member State in the African region stratified by the two age groups were obtained from World Bank data [15]. The empirical IFR was obtained from a study on serology-informed estimates of SARS-CoV-2 infection fatality rate (IFR) in Geneva, Switzerland and used as a reference population-based estimate [16]. The empirical IFR for this study was selected specifically because the population stratification matched the available population data from the World Bank for the analyses conducted herein (e.g. 65 years and above and those below 65 years of age). As of 03 January 2021, Switzerland was experiencing community transmission with 450 075 cumulative confirmed cases, including 7049 deaths, providing a 1.6% case fatality ratio (CFR) and a moderate cumulative incidence (814 per million population) [6]. As such the pandemic pattern in Switzerland is similar to that of most countries in the WHO African region. Based on the Switzerland study, the presumed age-adjusted SARS-CoV-2 IFR was computed to be 5.6% among those aged 65 years and above and 0.1% for those below 65 years.

Four parameters of interest were assessed, namely the total number of cases, the total number of missed cases, the detection rate and the cumulative incidence. The estimated total number of cases was defined as the total number of persons who were infected irrespective of whether these infections were detected and reported or not. The presumed age-adjusted IFR and the estimated total number of deaths in identified cases were used to compute the estimated total number of cases. The estimated total deaths in identified cases were defined as the sum of the total deaths among existing infections and the number of deaths yet to occur among current cases (obtained by applying the CFR to the number of cases in the last 14 days). The estimated number of missed cases refers to all those who were infected by the SARS-CoV-2 virus but who were not detected and reported. The other two parameters were derived estimates. These included the estimated cumulative incidence, which measured the actual extent of infection in the population based on the estimated total number of cases and the detection rate, which measured the proportion of cases detected among the total cases. Table 1 provides details on the formulae used to compute the four parameters as well as the presumed aged-adjusted IFR and the estimated total number of deaths in all cases identified.

**Table 1.** Indicators and formulae from the COVID-19 prevalence calculator tool [15]

Indicators	Formulae
Estimated total number of cases^a^	= [Estimated total deaths in all identified cases^b^] / [Estimated IFR^c^]
Estimated cases missed^d^	= [Estimated total number of cases] – [Observed cumulative cases]
Estimated detection rate (%)	= ([Observed cumulative cases] / [Estimated total number of cases]) * 100
Estimated cumulative incidence	= [Estimated total number of cases] / [Population]

a For the estimated number of cases, the IFR CIs were used considering a margin of error equal to 0.5 with 95% CI.

b The Estimated total deaths in all identified cases were obtained by adding the number of deaths yet to occur among current cases to the cumulative number of deaths reported. The number of deaths yet to occur among current cases was computed by multiplying the observed CFR by the number of new cases in the last 14 days for whom the definitive clinical outcome is not yet known. The number of cases in the past 14 days was obtained by deducting the total cumulative cases as of 14 days ago (17 December 2020) to the cumulative cases as of 31 December 2020.

c The Estimated IFR was obtained by adding the estimated IFR in population aged 65 years or above (% of the population aged ⩾65 years multiplied by the empirical IFR in the population aged ⩾65 years) to the estimated IFR in the population aged below 65 years (One minus the % of the population aged ⩾65 years multiplied by empirical IFR in those aged below 65 years).

d For the estimated cases missed, detection rate and cumulative incidence, the upper and lower estimates were obtained by applying the lower and upper estimated total number of cases.

### Inclusion and exclusion criteria

We included in our study all cases that were laboratory confirmed by reverse transcriptase-polymerase chain reaction (RT-PCR) test or antigen rapid diagnostic test, that were officially reported to the WHO Regional Office for Africa in accordance with the International Health Regulations (IHR 2005) [17] and the most recent WHO technical guidance [18].

In instances where official reports of laboratory-confirmed cases were not available for the studied period, the Member State was excluded from the analysis. In order to get an accurate estimation of the number of COVID-19 cases, Member States with an observed CFR below 0.5% were excluded in this analysis. A CFR below 0.5% may reflect poor recording and reporting of COVID-19 deaths, which would provide a less robust estimate of the true number of COVID-19 cases.

### Data analysis

We used the COVID-19 Prevalence Calculator method developed by Resolve to Save Lives [18, 19] for our analysis. The statistical underpinnings of the COVID-19 Prevalence Calculator method are based on the following assumptions: (1) the estimated number of SARS-CoV-2 cases includes all infected individuals, ranging from asymptomatic cases, pre-symptomatic cases and symptomatic cases to deaths and recovered patients; (2) the IFR is influenced by many factors, notably including the age distribution of the population, the prevalence of comorbidities and the availability of comprehensive case management strategies; (3) there is an observed lag of approximately 14 days on average between the onset of illness and death [18, 20]; and (4) cases in the past 14 days will have a similar CFR to those recorded before this time period.

Based on these assumptions, we computed the estimates of four outputs of interest from the COVID-19 Prevalence Calculator using the formulae described in Table 1 [18]. We used the regional line list of cases and deaths as of 31 December 2021 to generate the cumulative number of COVID-19 cases, the cumulative number of deaths, the number of deaths in the past 14 days (18 to 31 December 2021), the CFR of each country included in this study. We then calculated the number of deaths yet to occur among current cases by multiplying the CFR by the number of cases recorded in the past 14 days. For each country, the number of deaths yet to occur was added to the cumulative number of deaths in order to estimate the total number of deaths in all identified cases. This total was divided by the estimated IFR to get the mid-point estimate of the true number of cases. We divided the estimated number of cases by the population to estimate the cumulative incidence per hundred population. The difference between the estimated and observed number of cases gave the estimated number of cases missed. We divided the observed number of cases by the estimated number of cases to calculate the detection rate.

For each outcome of interest, we presented the mid-point estimates with a 95% confidence interval (CI). The upper and lower estimates of the CI were obtained considering a margin of error equal to 0.5 with 95% CI. We used R version 4.0.1 [21] to compute the four parameters of interest based on the COVID-19 Prevalence Calculator method and to plot the cumulative incidence by country, and ESRI 2017 ArcGIS Pro 2.1.0 [22] for mapping.

### Study validity

We intended to identify studies that could be used to test the validity of the tool but found few applicable studies. In the end, we assessed the validity of the tool by comparing our results with those from a representative population-based seroprevalence study from Spain [20]. The seroprevalence study was conducted between 27 April and 11 May 2020. We applied the number of cases and deaths in Spain as of 11 May 2020 and 14 days before, using WHO situation reports, to estimate the cumulative incidence using the COVID-19 Prevalence Calculator. Using the same empirical IFR, we got a cumulative incidence of 5.2% [95%CI: 4.3%–6.5%] compared to 5.0% [95% CI 4.7–5.4] from the seroprevalence survey.

## Results

### Study population

In accordance with the (IHR 2005) [17], all member states in the African region share their national situation reports on the COVID-19 pandemic with the WHO Regional Office for Africa. These reports include cases and deaths reported at a given point in time. As of 31 December 2020, the 47 countries of the WHO African region reported a total of 1 907 234 confirmed COVID-19 cases and 43 088 deaths (CFR = 2.3%). Four countries recorded a CFR below 0.5% and were excluded: Botswana (0.3%), Burundi (0.2%), Eritrea (0.2%) and Seychelles (0.0%). Tanzania had not reported new confirmed cases officially to the WHO since 07 May 2020 and was excluded from the analysis. A total of 42 countries were included in this study. These countries are heterogeneous in terms of population size and proportion of the population aged 65 years or above. The median population size was 12.5 million ranging from 215 056 in Sao Tome and Principe to 200.9 million in Nigeria. Seven countries had a population of less than 2 million including four island nations (Cape Verde, Comoros, Mauritius and Sao Tome and Principe). The median proportion of the population aged 65 years or above was 2.9% ranging from 1.9% in Uganda to 11.9% in Mauritius. Eight countries had more than 4% of their population aged 65 years or above: Eswatini (4.0%), Botswana (4.3%), Cape Verde (4.6%), Lesotho (4.9%), South Africa (5.4%), Algeria (6.5%), Seychelles (7.8%) and Mauritius (11.9%).

### Cumulative cases and deaths

As of 31 December 2020, a total of 1 889 512 confirmed cases and 43 020 deaths were reported by the 42 countries included in the study, representing 99.1% of cases and 99.8% of deaths in the African region. South Africa, Algeria, Ethiopia, Kenya and Nigeria accounted for 77.6% of cases and 85.0% of deaths. Three countries, Chad, Liberia and Mali, reported the highest CFR (4.9%, 4.6% and 3.8%, respectively). The lowest CFR was reported by Côte d'Ivoire (0.6%), Gabon (0.6%) and Guinea (0.6%).

### Estimated total number of COVID-19 cases compared with the reported number of cases

As of 31 December 2020, the estimated total number of COVID-19 cases in the 42 countries included in this study was 13 947 631 [95% CI: 13 334 620–14 635 502] against 1 889 512 reported laboratory confirmed cases, indicating that 13.5% (or 1 in 7) cases were detected. Six countries out of 42 had a detection rate above 30%, namely Cape Verde (36.6% (95%CI: 34.9,0%–38.3%)), Mauritius (40.0% (95% CI: 38.2%–41.9%)), Cote d'Ivoire (41.0 % (95% CI: 39.1%– 42.9%), Gabon (43.4% (95% CI: 41.4%–45.4%)), Ghana (43.5% (95% CI: 41.5%–45.6%)) and Guinea (43.9% (95% CI: 41.8%–45.9%)). Twenty-six countries out of 42 had a detection rate ranging between 10% and <30% and 10 countries below 10% (Table 2).

**Table 2.** Estimated total cases of SARS-CoV-2 infections and case detection rate by countries, as of 31 December 2020

Country	Cumulative cases	Cumulative deaths	CFR^a^	Estimated total cases (95% CI)	Estimated % of cases detected (95% CI)
Guinea	13 738	81	0,6	31 321 [29 944–32 866]	43.9 [41.8–45.9]
Ghana	54 930	335	0,6	126 132 [120 585–132 352]	43.5 [41.5–45.6]
Gabon	9571	64	0,7	22 037 [21 068–23 124]	43.4 [41.4–45.4]
Cote d'Ivoire	22 250	137	0,6	54 259 [51 873–56 935]	41.0 [39.1–42.9]
Mauritius	527	10	1,9	1316 [1258–1381]	40.0 [38.2;41.9]
Cape Verde	11 840	113	1	32 365 [30 942–33 961]	36.6 [34.9–38.3]
Mozambique	18 642	167	0,9	69 682 [66 618–73 119]	26.8 [25.5;28.0]
Namibia	24 545	208	0,8	92 145 [88 093–96 689]	26.6 [25.4–27.9]
Uganda	35 511	265	0,7	148 119 [141 606–155 424]	24.0 [22.8;25.1]
Rwanda	8383	92	1,1	40 892 [39 094–42 909]	20.5 [19.5–21.4]
Benin	3251	44	1,4	16 096 [15 388–16 890]	20.2 [19.2–21.1]
Central African Republic	4963	63	1,3	24 724 [23 637–25 943]	20.1 [19.1–21.0]
Comoros	765	9	1,2	4104 [3923–4306]	18.6 [17.8–19.5]
Madagascar	17 714	261	1,5	97 972 [93 663–102 804]	18.1 [17.2–18.9]
Ethiopia	124 264	1937	1,6	688 779 [658 488–722 748]	18.0 [17.2–18.9]
Lesotho	2577	50	1,9	15 098 [14 434–15 842]	17.1 [16.3–17.9]
Algeria	99 610	2756	2,8	631 621 [603 844–662 771]	15.8 [15.0–16.5]
Sao Tome & Principe	1014	17	1,7	6469 [6184–6788]	15.7 [14.9–16.4]
Congo	6200	100	1,6	40 052 [38 290–42 027]	15.5 [14.8–16.2]
Nigeria	87 510	1294	1,5	586 759 [560 955–615 697]	14.9 [14.2–15.6]
South Sudan	3558	63	1,8	24 499 [23 422–25 707]	14.5 [13.8–15.2]
Cameroon	26 848	448	1,7	186 148 [177 962–195 329]	14.4 [13.7–15.1]
Equatorial Guinea	5277	86	1,6	37 283 [35 644–39 122]	14.2 [13.5–14.8]
Guinea-Bissau	2447	45	1,8	17 477 [16 709–18 339]	14.0 [13.3–14.6]
Burkina Faso	6828	85	1,2	50 344 [48 130–52 827]	13.6 [12.9–14.2]
Kenya	96 458	1681	1,7	741 501 [708 892–778 071]	13.0 [12.4–13.6]
Togo	3633	68	1,9	28 593 [27 336–30 003]	12.7 [12.1–13.3]
South Africa	1 057 561	28 887	2,7	8 516 006 [8 141 498–8 935 998]	12.4 [11.8–13.0]
Senegal	19 140	410	2,1	164 931 [157 678–173 065]	11.6 [11.1–12.1]
Mauritania	13 642	324	2,4	119 520 [114 264–125 415]	11.4 [10.9–11.9]
Eswatini	9358	216	2,3	87 590 [83 738–91 910]	10.7 [10.2–11.2]
Zambia	20 725	390	1,9	201 070 [192 228–210 987]	10.3 [9.8–10.8]
Angola	17 553	405	2,3	194 149 [185 611–203 725]	9.0 [8.6–9.5]
Sierra Leone	2560	76	3	29 757 [28 448–31 224]	8.6 [8.2–9.0]
Zimbabwe	13 867	369	2,7	161 025 [153 944–168 967]	8.6 [8.2–9.0]
Malawi	6583	191	2,9	83 160 [79 503–87 261]	7.9 [7.5–8.3]
Gambia	3800	124	3,3	51 484 [49 220–54 023]	7.4 [7.0–7.7]
Democratic Republic of Congo	17 658	591	3,3	257 961 [246 617–270 683]	6.8 [6.5–7.2]
Liberia	1800	83	4,6	29 924 [28 608–31 400]	6.0 [5.7–6.3]
Niger	3208	102	3,2	53 126 [50 790–55 746]	6.0 [5.8–6.3]
Mali	7090	269	3,8	131 517 [125 733–138 003]	5.4 [5.1–5.6]
Chad	2113	104	4,9	50 624 [48 398–53 121]	4.2 [4.0–4.4]
Total	1 889 512	43 020		13 947 631 [12 730 416–14 635 502]	13.5 [12.9–14.2]

a CFR: Case fatality ratio.

**Fig. 1.**
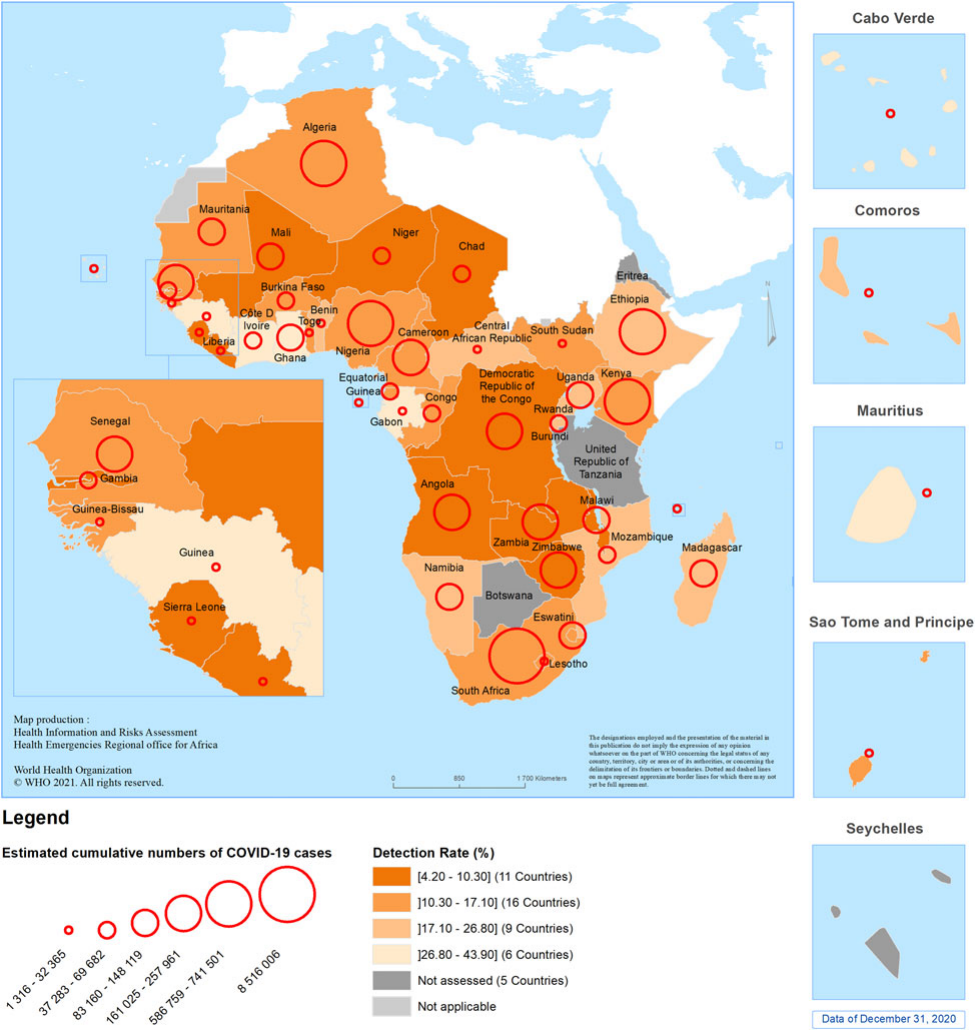
Geographical distribution of the estimated cumulative number of SARS-CoV2 infections and detection rate in 42 countries in the WHO African region, as of 31 December 2020.

Figure 1 presents the geographical distribution of the estimated cumulative number of SARS-CoV-2 infections and detection rate as of 31 December 2020 by country in the African region.

### Estimated cumulative incidence proportion of SARS-CoV-2 infections

**Fig. 2.**
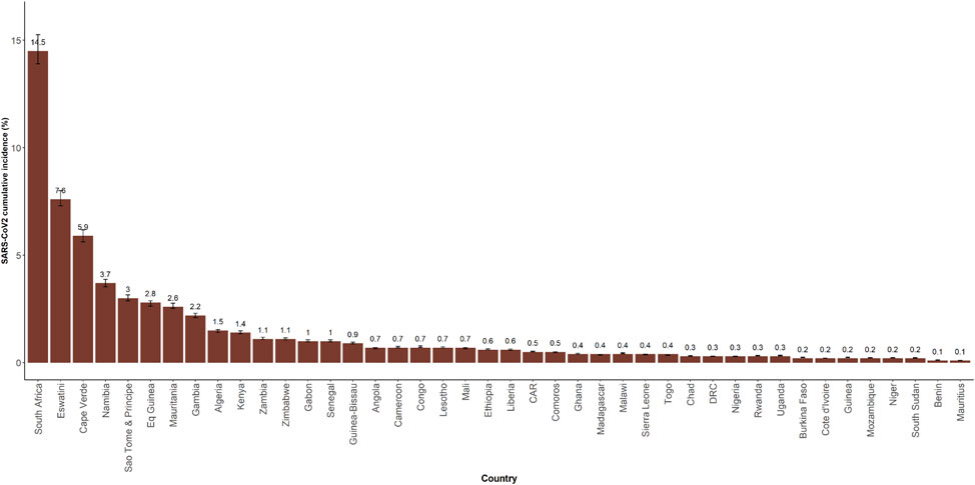
Estimated cumulative incidence of SARS-CoV-2 infection by country in the WHO African region, as of 31 December 2020.

As of 31 December 2020, the cumulative incidence of SARS-CoV2 infections in the 42 countries of the African region was estimated at 1.38% [95% CI: 1.31%–1.44%]. The highest cumulative incidence estimates were seen in South Africa (14.5% (95% CI: 13.9%–15.2%)), Eswatini (7.6% (95% CI: 7.3%–8.0%)), Cape Verde (5.8% (95% CI: 5.6%–6.2%)), Namibia (3.7% (95% CI: 3.5%–3.9%)) and São Tomé & Príncipe (2.9% (95% CI: 2.9%–3.1%)). The lowest cumulative incidence estimates were reported in Mauritius (0.1% (95% CI: 0.099%–0.11%), Benin (0.13% (95% CI: 0.13%–0.14%)); Cote d'Ivoire (0.21% (95%CI: 0.20%–0.22%)), South Sudan (0.22% (95% CI: 0.21%–0.23%)), Niger (0.23% (95% CI: 0.22%–0.25%)) and Mozambique (0.23% (95% CI: 0.21%–0.24%)) (Fig. 2).

## Discussion

### Methods and tools for estimating the cumulative incidence of SARS-CoV-2 infection

As the pandemic continues to evolve, the tried and tested approaches that are being implemented by Member States of the WHO African region require innovative use of data to help track progress and assist in evidence-based decision making. Several pandemic performance metrics have been proposed to track the evolution of the pandemic and help make timely decisions in spite of the uncertainty surrounding the management of the pandemic. These include but are not limited to the observed number of new cases and deaths per time unit, the observed attack rate, the number of tests performed per 10 000 population and the testing positivity rate [23]. Estimating the true number of cases and the cumulative incidence of SARS-CoV-2 infection in each country in the WHO African region may help each country adjust its public health and social measures based on evidence, inform national and regional resource allocation and contribute to better provision of technical support and strategic guidance by the WHO Regional Office for Africa.

The reported number of confirmed cases and deaths as of 31 December 2020 in Member States of the WHO African Region are based on data that was made available in a context of a limited supply of laboratory tests in several countries, possibly limited laboratory capacities particularly at provincial and district levels and use of testing strategies that are narrowed and targeting specific groups of individuals [4, 7]. This has made it difficult to ascertain the true extent of the pandemic in the region. The region's large youth population may also lead to more infections, most being asymptomatic or mild and undetected [8]. Addressing this challenge ten-plus months into the pandemic requires the Member States to conduct serological testing of a representative random sample of the population in order to not only determine the extent of seroprevalence, but also to identify hotspots, groups most at-risk and contribute to informing public health measures and control strategies [9, 24, 25]. The already stretched human resources, scarcity of financial resources as well as competing priorities in supporting fast evolving and changing response operations prevent almost all countries in the region from conducting such studies in the general population. A literature review up to 14 August 2020 did not find any publication related to population-based SARS-CoV2 serosurveys from sub-Saharan African [26]. There are however reports of countries such as Kenya and Malawi conducting SARS-COV-2 seroprevalence surveys among blood donors and health workers, respectively [27, 28].

The COVID-19 Prevalence Calculator tool [18] developed by Resolve to Save Lives [19] appears to be an easy-to-use tool for estimating the SARS-CoV2 cumulative incidence, based on the reported cumulative number of cases and deaths, as well as empirical IFR from the available literature.

### Estimated number of SARS-CoV-2 infections

In this study, our estimated number of SARS-CoV-2 infections is far lower than the early projection made by Cabore *et al*. [5] of 223 million cases by the end of 2020. The modelling in Cabore *et al*. [5] assumed widespread community transmission in all countries of the region at an early stage of the pandemic. The more gradual change in disease transmission pattern from no cases, sporadic cases, a cluster of cases, to community transmission, implementation of public health and social measures and adherence to thesemeasures were not taken into consideration in the model. Early implementation of non-pharmaceutical interventions such as lockdowns may have delayed the spread of the pandemic in the region and resulted in lower numbers of cases reported than previously projected [29].

### SARS-CoV-2 cumulative incidence and detection rate

Beyond the cumulative number of cases detected, it is critical for each country affected by the COVID-19 pandemic to have an estimate of the cumulative incidence of SARS-CoV-2 to understand the extent to which its population has been infected. In this study, our estimate of the cumulative incidence of SARS-CoV-2 infection in the African region was 1.38%, ranging from 0.1% in Mauritius and Benin to 14.5% in South Africa. Recent surveys conducted in Kenya in blood donors [27] estimated the IgG antibodies seroprevalence at 5.6% [95% CI: 4.8%–6.5%] which was higher than the estimated cumulative incidence as 31 December 2020 in the general population in our study. This difference could be partly explained by the fact that in the Kenyan study, blood donors included were aged 15–64 years with 89% aged below 45 years [27]. This may have overestimated the seroprevalence with most SARS-COV-2 infections occurring in a young population [30]. In Malawi, a study by Chibwana *et al*. [28] estimated the SARS-CoV2 IgG antibodies seroprevalence in healthcare workers at 12.3% [95% CI: 8.2%–16.5%] compared with 0.45% [0.43%;0.47%] estimated cumulative incidence in our study. This could partly be explained by the higher exposure of frontline healthcare workers to the virus [31], resulting in higher seroprevalence than in the general population.

Estimation of the SARS-CoV-2 cumulative incidence provides a proxy of the proportion of the population who have been exposed to the virus where widespread serosurveys are not yet feasible, though questions remain about the extent and duration to which infected individuals may be protected following recovery. Our results suggest that the vast majority of the population in the African region, including in South Africa, is still immunologically naïve to SARS-CoV-2 ten months into the pandemic, and therefore population immunity is insufficient to avoid potential subsequent waves of the pandemic. Indeed, assuming an estimated reproduction number of 3 for SARS-CoV-2, the immunity threshold is approximatively 67% [32]. This suggests the pandemic will smoulder on until the proportion of individuals with acquired immunity to SARS-CoV-2 in the population exceeds 0.67 [32], especially through vaccination [33].

This study estimated that only one SARS-CoV-2 case in seven is detected, with the detection rate ranging from 4.2% in Chad to 43.9% in Guinea. This low detection rate may be a result of limited testing capacity, testing strategies focusing on people with suggestive symptoms while the majority of infected people are asymptomatic, a large youth population with less severe disease presentation and relatively low rates of obesity across the region in comparison to many Western countries [4, 8]. Further, the gap in detection is not uniform across the region, so while some countries have high detection rate estimates, low estimates across the rest of the region lower the overall detection rate estimates. The use of the COVID-19 Prevalence Calculator tool could help each country in the WHO African region to monitor the estimated cumulative incidence of SARS-CoV-2 infection at the sub-national level to adjust its tailored response strategy.

The low detection rate of COVID-19 cases reported in this study has provided evidence of suboptimal COVID-19 surveillance systems in place in most countries in the WHO African region for early detection and rapid isolation of cases. Case detection is premised on a strong surveillance system that can identify suspected cases including asymptomatic and pre-symptomatic cases [34]. In addition to limited testing capacity [29], Nachega JB *et al*. [35] found the overwhelming workload of contact tracing and case detection by healthcare workers among the main challenges of the COVID-19 response in Nigeria, Rwanda, South Africa and Uganda. The clinical pattern of COVID-19 cases in Africa is that most cases are asymptomatic or mildly symptomatic [34]. This, coupled with a surveillance strategy that relies mainly on clinical criteria [17] to enrol suspected cases for SARS-CoV-2 testing and underperforming contact tracing, is also contributing to the low detection of COVID-19 cases. Additionally, as result of fear of COVID-19 and movement restrictions during lockdowns, utilisation of basic health services declined [36], which also contributed to low detection of COVID-19 cases.

### Limitations

The interpretation of the results of this study should take into consideration a number of limitations.

The COVID-19 Prevalence Calculator tool uses the observed CFR, the number of deaths cumulatively and in the past 14 days, the proportion of population aged 65 years or above and an empirical IFR from a population based-study to compute estimates of the true number of cases, the cumulative COVID-19 incidence and the detection rate.

All deaths are unfortunately not being captured by the surveillance systems in place in most countries in the WHO African region. Karlinsky A and Kobak D [37] reported an increase of 27% in annual mortality in 2020 in South Africa, highlighting the possible underreporting of deaths. A post-mortem surveillance study in Zambia [38] detected SARS-CoV-2 in 15.9% of deaths, most of which occurred in people not tested for SARS-CoV-2 before death. Underreporting of deaths may result in underestimation of the CFR and therefore an underestimation of the true number of cases and overestimation of the detection rate [39].

We used an empirical IFR from Switzerland, a country with a better case management capacity than most countries of the region. The surveillance and case management systems in place in countries in the WHO African region mean that the majority of cases detected have a severe and critical disease, which may result in a higher CFR than currently reported and therefore an underestimation of the true number of cases and overestimation of the detection rate.

The distribution of ages within the two defined age groups (below 65 years, 65 years and above) are quite broad and highly variable between countries, and this may affect both the estimates and the comparability of the resulting estimates between countries.

Underreporting of deaths and lower case management capacity than in the country that provided the empirical IFR may limit the external study validity. The COVID-19 Prevalence Calculator method was designed for the general population and may not be valid for estimating the COVID-19 prevalence in specific groups as age distribution and CFR may be different.

## Conclusion

The African region is the least affected by the COVID-19 pandemic globally. Due to limited capacities and funding, very few countries have performed population-based serosurveys using antibody tests, leaving the true extent of SARS-CoV-2 infections unknown. Applying the COVID-19 Prevalence Calculator tool helped to estimate the prevalence of SARS-CoV-2 for each country in the region with a CFR of at least 0.5%. The low detection rate for most of the countries in the African region suggests the need to adjust testing strategies to better align with the observed patterns of the pandemic and better understand the magnitude of infections. This highlighted the need for continued efforts to strengthen disease surveillance in countries to ensure early detection and rapid isolation of all COVID-19 cases including those with no or mild symptoms. Further, in the absence of population-based serosurveys, it may be useful for the WHO Regional Office to provide technical assistance to the Member States in using the COVID-19 Prevalence Calculator to estimate the SARS-COV-2 cumulative incidence and detection rate at the subnational level in order to implement localised, tailored public health and social response measures.

## Data

The datasets generated and/or analysed during the current study are available from the corresponding author on reasonable request.
